# Orally administered live BCG and heat-inactivated *Mycobacterium bovis* protect bison against experimental bovine tuberculosis

**DOI:** 10.1038/s41598-025-88176-0

**Published:** 2025-01-30

**Authors:** Nirajan Niroula, Priya Ghodasara, Nelson Marreros, Bailey Fuller, Haley Sanderson, Slim Zriba, Stew Walker, Todd K. Shury, Jeffrey M. Chen

**Affiliations:** 1 Parks Canada Agency, Government of Canada, Gatineau, Quebec, Canada; 2 Vaccine and Infectious Disease Organization (VIDO), University of Saskatchewan, Saskatoon, SK Canada; 3 School of Agricultural, Forest and Food Sciences, Bern University of Applied Sciences, Canton Berne, Switzerland; 4 McKenzie Veterinary Services, Victoria, BC Canada

**Keywords:** Bison, Bovine TB, Heat Inactivated Vaccine, Live BCG, DIVA, Immunology, Microbiology, Diseases, Pathogenesis

## Abstract

Bovine tuberculosis (BTB) is an infectious disease of livestock and wildlife species that is caused by pathogenic members of the *Mycobacterium tuberculosis* complex such as *Mycobacterium bovis*. Due to the introduction of *M. bovis*-infected bison in the 1920s, BTB is now endemic in wood bison (*Bison bison athabascae*) population within the Wood Buffalo National Park (WBNP) in northern Canada. This disease poses a grave threat to the long-term survival of this ecologically and culturally important species and has the potential to cause zoonotic TB and spill over to BTB-free livestock and other bison herds that live in the surrounding areas. Thus, effective BTB control strategies in WBNP bison are urgently needed. To this end, we aerosol challenged young bison with different doses of virulent *M. bovis* and observed disease-associated delayed-type hypersensitivity, gross lung and lymph node pathology and histopathology, as well as *M. bovis* burden in target organs, thus confirming the establishment of BTB in challenged animals. We then assessed the safety and efficacy of oral live BCG versus oral heat-inactivated *M. bovis* (HIMB) given in a homologous prime-boost regimen in bison. While both BCG and HIMB offered protection against BTB, BCG-treated bison thrived more, presented with fewer lung lesions at necropsy and lower burden of virulent *M. bovis* than HIMB-treated animals. Strikingly, oral HIMB induced almost no delayed-type hypersensitivity to intradermal tuberculin while oral live BCG induced very low sensitivity to tuberculin in bison, indicating their potential as DIVA (differentiating infected from vaccinated animals) vaccines for use in this important wildlife species.

**Supplementary Information:**

The online version contains supplementary material available at 10.1038/s41598-025-88176-0.

## Introduction

Bovine Tuberculosis (BTB) is a globally distributed and highly contagious bacterial infection that affects livestock, wild ungulates and a variety of other mammals^1^. It is caused by pathogenic members of the *Mycobacterium tuberculosis* complex (MTBC) like *M. bovis* and also a source of zoonotic infections in humans^1,2^. BTB is mainly transmitted in aerosols containing the TB bacteria and sometimes also through ingestion of contaminated milk and meat^1^. As such, the disease affects the lungs and lymph nodes associated with the respiratory tract such as the retropharyngeal, tracheobronchial and mediastinal lymph nodes^1,3^. *M. bovis*-containing lesions in the lungs and lymph nodes can undergo necrotic degradation over time, leading to active symptomatic BTB disease and transmission. Active BTB is also marked by fever, weight loss, diarrhea and occasionally respiratory distress, and ultimately results in the death of diseased animals^1,3,4^.

In some jurisdictions, control and even eradication of BTB in livestock has only been possible through disease testing, surveillance and culling of BTB-positive animals^5,6^. However, once BTB becomes established in free-ranging wildlife hosts, it becomes challenging to eradicate^7^. Indeed, numerous wildlife reservoirs of BTB exist and have been implicated in spillover of disease to domesticated animals^1^. Well described examples include white-tailed deer, bison and elk in North America, badgers in the UK, wild boar in Spain and Portugal, brushtail possums in New Zealand, and a variety of iconic wildlife species such as the African buffalo and white rhinoceros in South Africa^8–13^. In Canada specifically, BTB has been reported in elk, moose, mule deer, wolves and bison^8,10^. Indeed, the disease is currently endemic in several herds of wood bison (*Bison bison athabascae*), a culturally and ecologically important keystone species in and around Wood Buffalo National Park (WBNP) in northern Canada. Records suggest BTB was likely introduced into the area during the 1920s, via the inadvertent translocation of North American plains bison (*Bison bison bison*) infected with *M. bovis* that had spilled over from diseased cattle^8,14^. As the last remaining wildlife reservoir of BTB in Canada, proposed plans to eliminate the disease from WBNP wood bison through depopulation in the 1990s were never implemented due to concerns from the general public and indigenous communities^15^. Together with brucellosis, BTB remains a major threat to the conservation of wood bison in Canada, and two herds have recently been assessed to face imminent danger to their recovery^14^. As such, more effective and socially acceptable BTB control strategies are needed to tackle the threat of this disease to wood bison in WBNP. While oral vaccines have proven to be effective for BTB control in European badgers^16–19^, brush-tailed possums^12^, wild boar^20–23^and deer^24,25^, this approach has not been explored with bison.

Thoen and colleagues were the first to study BTB pathogenesis and transmission under an experimental setting in bison^26^. They administered either live *M. bovis*, heat-killed *M. bovis* or saline to groups of year-old American bison (*Bison bison*) via the intratracheal route and monitored them for up to a year post-challenge. At 30 days post-challenge, they also introduced and co-housed Jersey cattle calves (*Bos taurus*) with the live *M. bovis*-infected bison^26^. While animals administered live and heat-killed *M. bovis* produced tuberculin-specific serum antibodies, only bison challenged with live *M. bovis *became significantly positive for T-cell proliferation in response to tuberculin^26^. Additionally, only bison challenged with live *M. bovis* developed visible tuberculous lesions in the lungs and lymph nodes at necropsy. Concordantly, microscopic granulomas were seen in lung tissues and viable tubercle bacilli were recovered from animals challenged with live *M. bovis*^26^. Finally, a third of the cattle calves that were co-housed with live *M. bovis*-infected bison became sensitized to tuberculin although visible tuberculous lesions were not detected nor were live *M. bovis* recovered from the co-housed calves^26^. Although this pioneering study clearly showed that bison are susceptible to BTB and can transmit the disease to cattle, gaps in knowledge remained which we sought to address here. In the first part of our study, we challenged healthy young bison via the aerosol route with defined doses of a virulent isolate of *M. bovis* endemic to WBNP designated *M. bovis* 0286 and monitored them for clinical signs of BTB progression as well as pathological and immunological changes associated with infection. In the second part of our study, we examined in bison the immunogenicity and protective efficacy of orally administered Bacillus Calmette-Guerin (BCG), a live attenuated vaccine strain that was originally derived from virulent *M. bovis* a century ago and is widely used in humans^27^, and orally administered heat-inactivated *M. bovis* 0286 in a prime-boost regimen.

## Results

### Aerosol *M. bovis* challenge of bison

#### Clinical signs

**Fig. 1:**
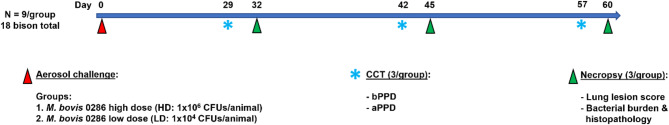
Trial schematic of bison aerosol challenged with high and low inoculum doses of virulent *M. bovis* 0286. 18 bison were randomly distributed into 2 groups of 9 animals each. One group was challenged via the aerosol route with an estimated 10^6^ CFUs per animal while the other was challenged with an estimated 10^4^ CFUs per animal. At days 29, 42 and 57 post-challenge, 3 animals per group were randomly selected and administered the CCT test. 72 h later, changes in skin thickness were measured and the animals were euthanized and necropsied.

**Fig. 2:**
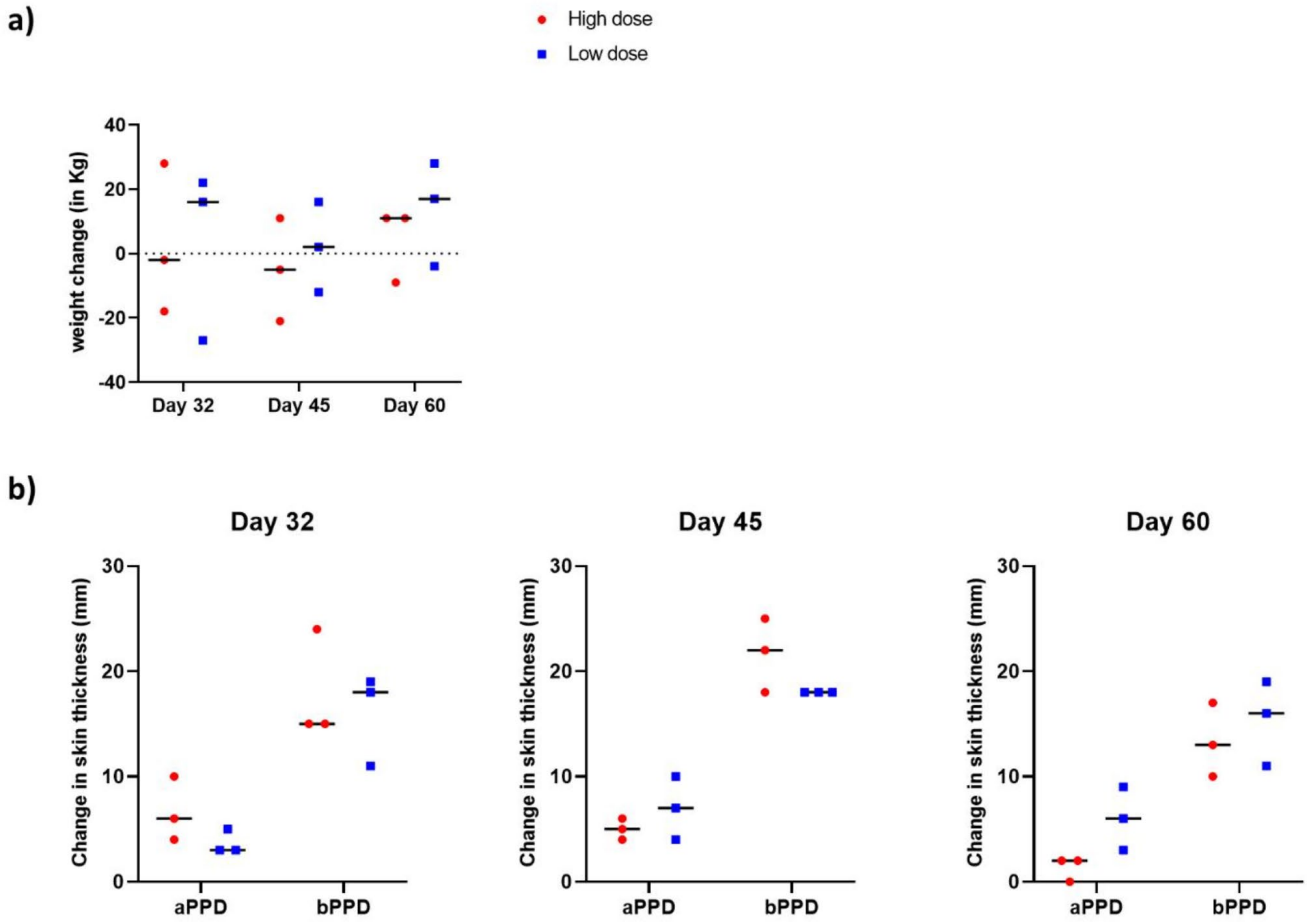
Weight changes and tuberculin sensitivity in bison challenged with high and low inoculum doses of *M. bovis* 0286. (**a**) Scatter dot-plot representing weight changes (in Kg) in bison calves where each dot indicates the weight change of an individual animal, and the horizontal bar indicates the median. The data points below the dashed line represent animals that lost weight during the course of the trial. (**b**) Comparative cervical skin test of animals euthanized on day 32, 45 and 60 post challenge. The result is expressed as a change in skin thickness (in mm) 72 h after intradermal injection of avian and bovine tuberculin. Each dot represents an individual animal, and the horizontal bar is the median.

Over the duration of the first trial (Fig. 1), none of the bison challenged with high dose (HD) or low dose (LD) *M. bovis* 0286 showed signs of active BTB such as fever or respiratory distress. However, we did notice that more animals challenged with HD *M. bovis* failed to thrive by not gaining as much weight as those challenged with LD *M. bovis* (Fig. 2a). Moreover, five out of the nine animals in the HD *M. bovis* group and three out of nine in the LD *M. bovis* group actually lost weight by their respective euthanasia times.

### DTH response

Delayed-type hypersensitivity (DTH) to *M. bovis* purified protein derivative (bPPD) or bovine tuberculin and *M. avium paratuberculosis* purified protein derivative (aPPD) or avian tuberculin in *M. bovis*-infected bison was examined by comparative cervical skin-test (CCT). A positive CCT result for *M. bovis* infection is indicated if the difference in skin thickness between bPPD and aPPD injection sites 72 h post-administration is more than 4 mm^1,28^. Following these metrics, all animals tested positive when the skin test was administered and read before their respective euthanasia times at 32-, 45- and 60-dpc (Fig. 2b). Statistically significant differences in skin thickness between the HD and LD *M. bovis* animals were not observed (Fig. 2b). These results are consistent with the tuberculin skin-test findings in the bison-*M. bovis* infection study of Thoen and coworkers^26^.

### Gross Pathology

**Fig. 3:**
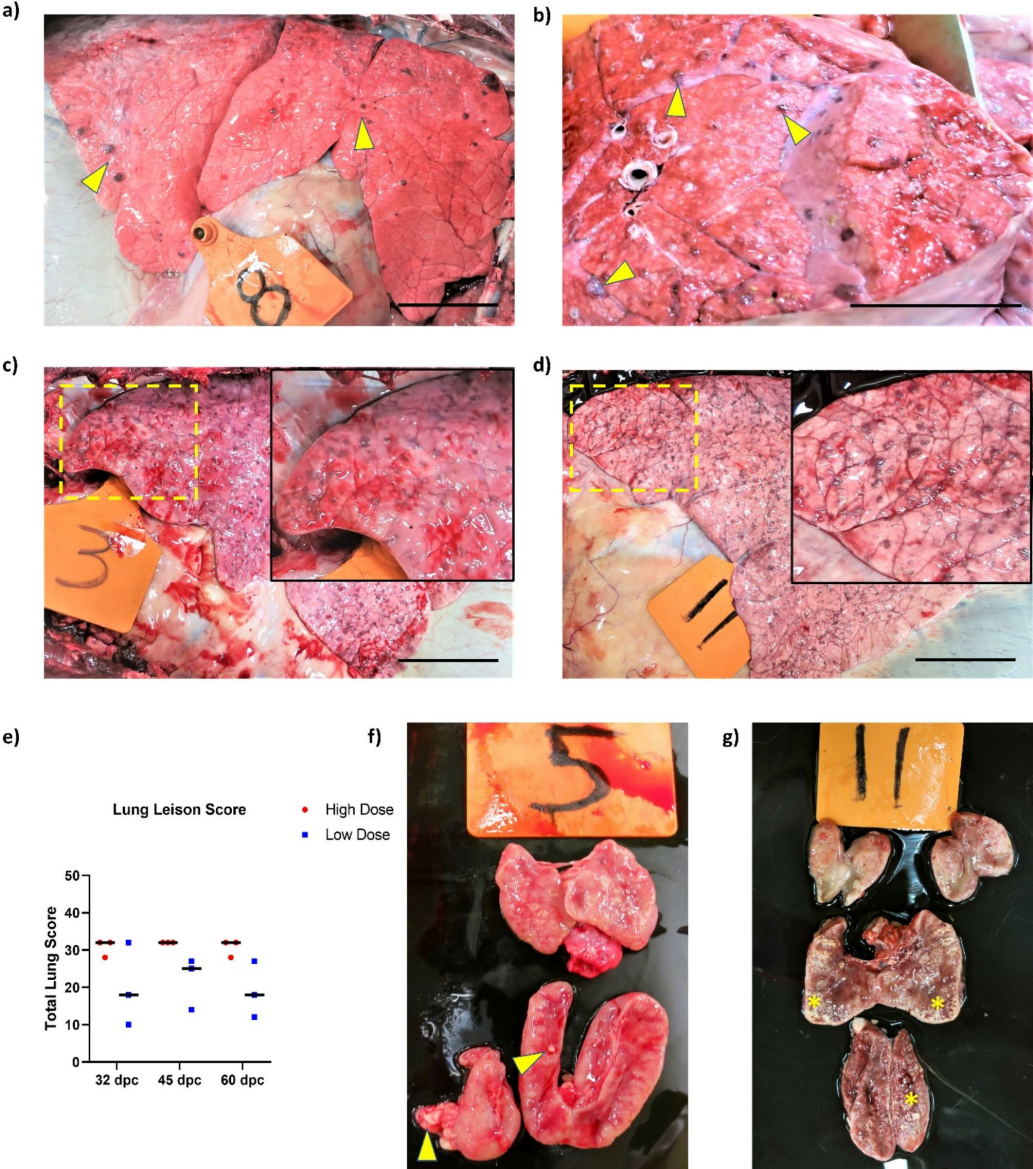
Gross findings at necropsy of bison challenged with high and low inoculum doses of *M. bovis* 0286. (**a**) Lungs of bison calves challenged with a low dose *M. bovis* and euthanized 32 days later presented with fewer but distinct granulomatous lesions (arrow heads). (**b**) Cut section of the lungs showing circular TB granulomatous lesions of different sizes (arrow heads). (**c**) Lungs of bison calf challenged with a high dose of *M. bovis* shows numerous tuberculous lesions on its surface at 32 and (**d**) at 60 days post-challenge. Large punctate, gray to reddish-brown lesions is visible on the inset. (**e**) Scatter dot-plot representing gross lung lesion score in bison calves challenged with a high and a low dose of *M. bovis* and euthanized at 32-, 45- and 60-days post-challenge. Each dot indicates total lung lesion score of individual animals and the horizontal bar is the median. (**f**) Lymph nodes of bison calves euthanized at 30 days post challenge had few lesions (arrow heads) compared to the calves euthanized at 60 days post challenge (**g**), which were presented with extensive lesions on the entire surface. The edge of the mediastinal and tracheobronchial LNs were presented with areas of caseous necrosis (asterisks). Scale bar = 75 mm.

Tuberculous lesions were found in the lungs and the associated lymph nodes (LNs) of all animals in both HD and LD *M. bovis* groups at necropsy. Pulmonary lesions ranging in size from 2 to 10 millimeters (mm) appeared as nodular lesions on the pleural surfaces (Fig. 3a, c and d). And visual examination of sectioned lung tissues often revealed circular granulomatous lesions distributed uniformly on the inner surfaces (Fig. 3b). Overall, we observed that animals with LD *M. bovis* (Fig. 3a) presented with fewer lung lesions than those challenged with HD *M. bovis* whose lesions were spread out uniformly in all lung lobes, that often coalesced and were challenging to count (Fig. 3c and d). Gross lung lesions in each lobe were quantified using a scoring system based on the number and size of granulomas^29,30^. The cumulative score gives the gross lesion score for each lung and was used to assess disease severity in each animal. Most animals challenged with HD *M. bovis* presented with numerous lesions on the pleural surface mostly with scores of 4 per animal while animals challenged with LD *M. bovis* presented with lesions scoring between 1 and 4 per animal. Moreover, the median lung lesion scores of HD *M. bovis* animals were higher than LD *M. bovis* animals at all three necropsy timepoints post-challenge (Fig. 3e).

Pulmonary draining LNs like the mediastinal and tracheobronchial LNs were positive for lesions in most of the challenged animals with some of the HD *M. bovis* challenged animals presenting with more enlarged LNs. Changes over time post-challenge in the thoracic LNs of HD *M. bovis* animals were also observed – while animals examined at 32-dpc had few lesions (Fig. 3f), those examined at 60-dpc had more tuberculous lesions with the inner surface of the organ filled with coagulating necrotic material throughout the cortex and sinuses (Fig. 3g).

### Histopathology

**Fig. 4:**
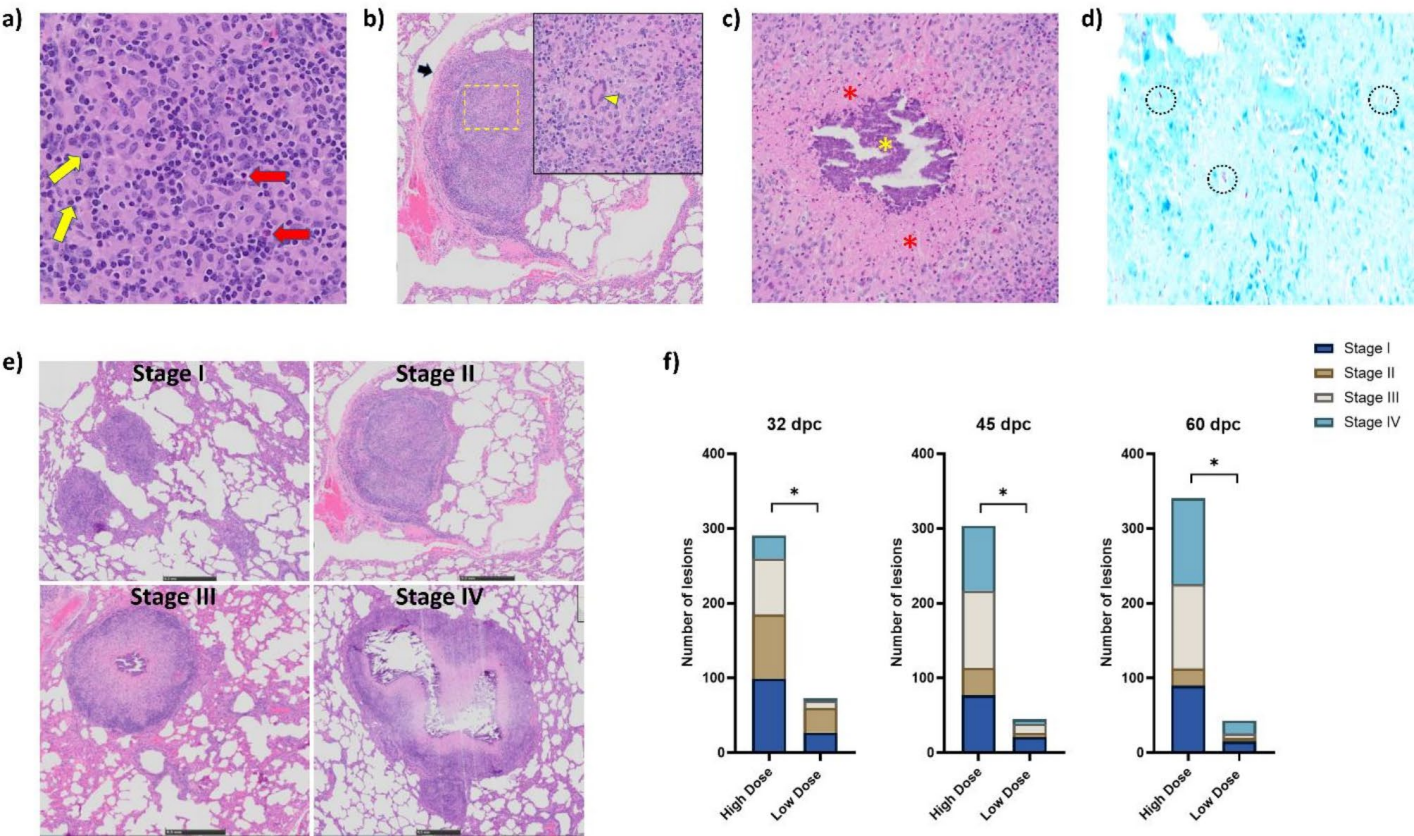
Histopathological characteristics of lung tissues from bison challenged with high and low inoculum doses of *M. bovis* 0286. (**a**) Aggregation of different immune cells to form a core of the early-stage granuloma. Epithelioid macrophages can be seen in abundance, while fewer lymphocytes (yellow arrows), and neutrophils (red arrows) are also observed. (**b**) Pulmonary granuloma with inner core of cellular aggregates surrounded by fibrous capsule (black arrow). Inset shows the Langhans type multinucleated giant cell (yellow arrowhead). (**c**) The core of a mature granuloma with the formation of amorphous zone (red asterisks) due to caseous necrosis that is being replaced by mineralization (yellow asterisks). (**d**) Ziehl-Neelsen staining of bison lungs with acid-fast *M. bovis* (encircled). (**e**) Histopathology of *M. bovis* infected bison lung tissues describing four stages of TB granuloma. Scale bar on each image is 0.5 mm. (**f**) Number and stages of microscopic granulomas observed in lung samples of bison calves. A statistically significant difference was observed between the high dose and a low dose challenged groups at all 3 timepoints post-challenge (P value < 0.05, Mann Whitney U Test).

Sections prepared from the lung tissues of all bison presented with microscopic granulomatous lesions consistent with BTB infection, irrespective of the challenge dose. Thickening of lung parenchyma was observed in most animals likely as a result of what appears to be macrophages, lymphocytes, and neutrophils infiltration. Well organized microscopic granulomas consisting of an inner core of inflammatory cellular aggregates such as epithelioid macrophages, foamy macrophages and multinucleated giant cells could be observed (Fig. 4a and b). These structures were often found to be surrounded by a mantle of lymphocytes encapsulated by fibrous capsules (Fig. 4b). In advanced stage microscopic granulomatous lesions, necrosis of infected cells was found to occur within the core of the lesion, leading to the formation of an amorphous zone that subsequently mineralized (Fig. 4c). Ziehl-Neelsen (ZN) staining also revealed the presence of multiple acid-fast mycobacteria in at least one lung tissue section from each animal (Fig. 4d). Using a previously described classification system of microscopic granulomatous lung lesions that stratifies them into 4 stages (I to IV) (Fig. 4e)^31–33^, statistically significant differences in the total number of lesions and distinct proportions of the different stages therein were noted in HD and LD *M. bovis* challenge groups at each of the three necropsy timepoints post-challenge (Fig. 4f). For instance, at 32-dpc, a total of 291 granulomas were observed in the lung sections of HD *M. bovis-*challenged bison while only 73 granulomas were observed in LD *M. bovis* animals. Granuloma numbers increased to a total of 341 in lung sections from HD *M. bovis* animals while a decrease to 43 was observed in LD *M. bovis* animals by 60-dpc. Notably, number of stage IV granulomas increased over time in both the HD and LD *M. bovis*-challenged bison, suggesting the animals were likely on their way to developing active BTB disease as stage IV lesions are often indicative of progressive disease. Concomitantly, the number of stage II lesions was seen to decline over time post-challenge.

### Bacterial burden

**Fig. 5:**
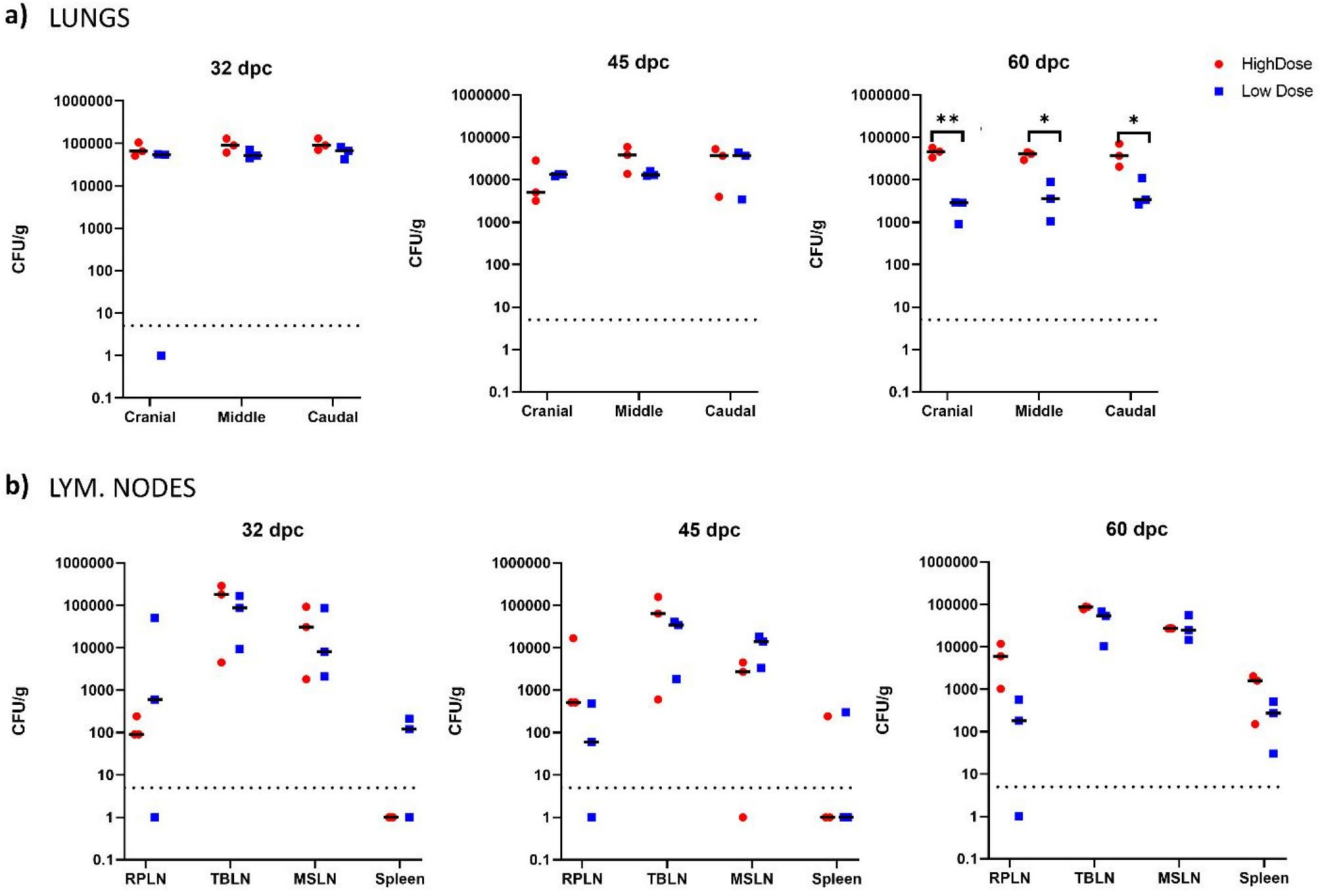
Bacterial burden in lung and lymphoid tissues from bison challenged with high and low inoculum doses of *M. bovis* 0286. Each dot in the graph represents CFU/g of tissue from an individual animal and the horizontal bar represent the median. The dashed line parallel to x-axis indicates the limit of detection. (**a**) The upper panel shows the burden in different lung lobes of animals euthanized at 32-, 45- and 60-days post challenge. (**b**) The lower panel shows the burden in lymphoid tissues of the same animals. Statistical analysis using unpaired T-test where ** indicates significance at *p* < 0.01 and * indicates significance at *p* < 0.05.

Viable *M. bovis* 0286 was recovered from the lungs and lymphoid tissues of all animals irrespective of the challenge dose and time post-challenge. At 32- and 45-dpc, the average bacterial burdens in the cranial, middle, and caudal lobes of the lungs were found to be similar between the HD and LD *M. bovis* groups (Fig. 5a). However, by 60-dpc the bacterial burden had dropped significantly by at least 1-log in the same lung lobes of LD *M. bovis* bison compared to HD *M. bovis* animals.

Regardless of challenge dose, *M. bovis* burden was found to be higher in the pulmonary draining LNs such as the tracheobronchial and mediastinal LN than in the retropharyngeal LN and spleen at 32-, 45- and 60-dpc (Fig. 5b). However, by 60-dpc bacterial burden in the retropharyngeal LNs and spleens of HD *M. bovis* animals was higher than in LD *M. bovis* animals (Fig. 5b).

## Protective efficacy of oral BCG and heat-inactivated ***M. bovis*** in bison

### Clinical signs

**Fig. 6:**
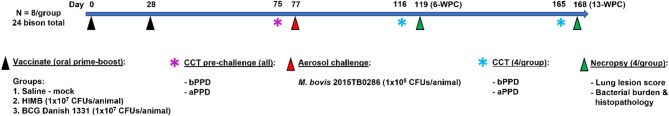
Trial schematic assessing protective efficacy of oral live BCG and oral heat-killed *M. bovis* against BTB in bison. 24 bison were randomly distributed into 3 groups of 8 animals each. One group was mock vaccinated with saline, the second group with an estimated 10^7^ CFU of HIMB and the last group with an estimated 10^7^ CFU of live BCG via the oral route. 28 days later, the immunizations were repeated for a prime-boost regimen. 75 days after the first dose of vaccinations, the CCT test was administered and 72-hours later changes in skin thickness measured. Thereafter all animals were challenged via the aerosol route with live *M. bovis* 0286 at an estimated 10^5^ CFUs per animal. At 116- and 165-dpc, 4 animals per group were randomly selected and administered the CCT test. 72 h later, changes in skin thickness were measured and the animals were then euthanized and necropsied.

**Fig. 7:**
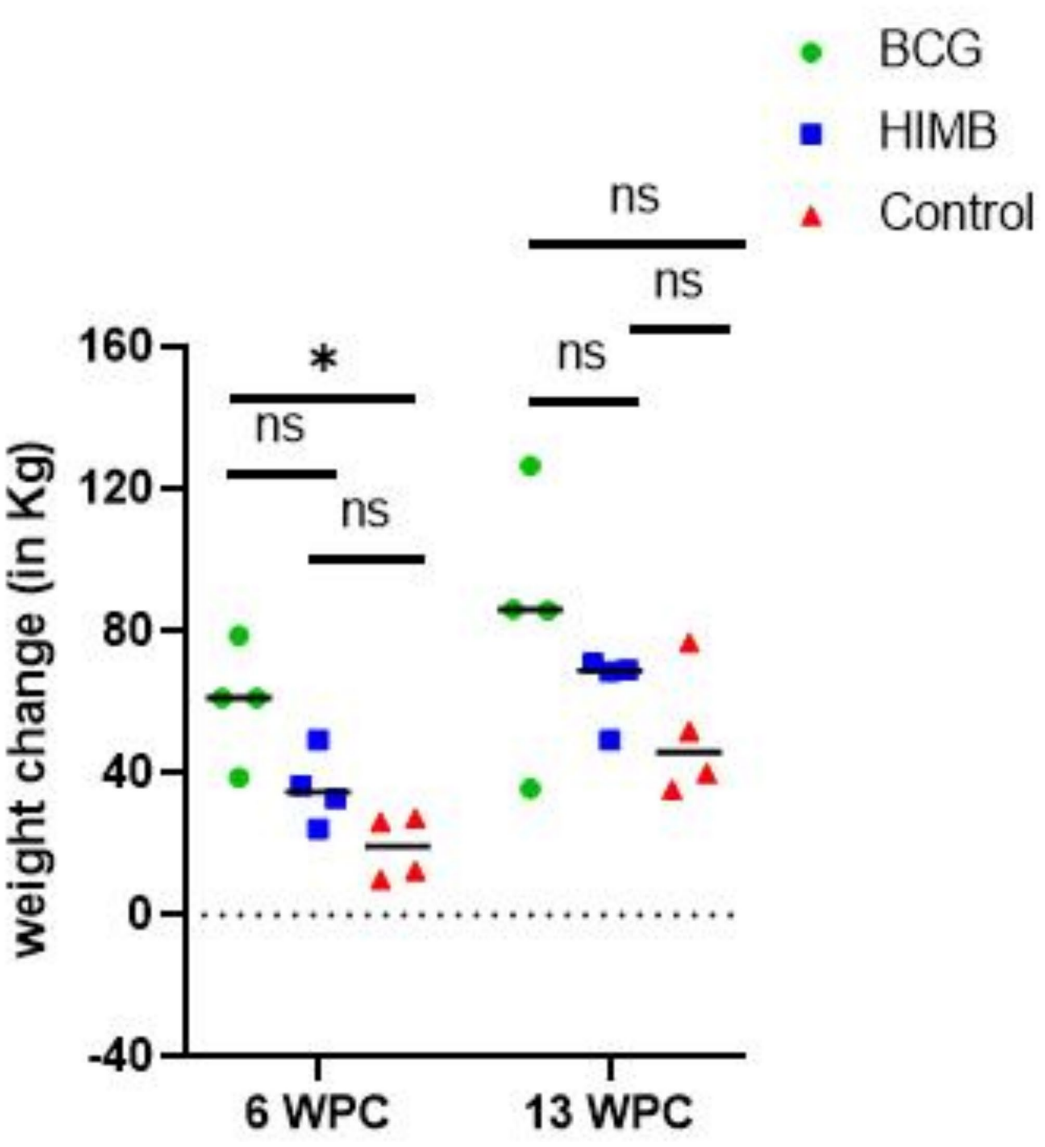
Weight changes in vaccinated vs. unvaccinated bison after challenge with *M. bovis* 0286. Scatter dot-plot representing weight changes (in Kg) in bison calves where each dot indicates the weight change of an individual animal, and the horizontal bar indicates the median. The data points above the dashed line represent animals that gained weight during the trial. A significantly higher weight gain was observed in calves vaccinated with BCG compared to the control at 6 wpc (Kruskal Wallis test with Dunn’s multiple comparison, *p* < 0.05).

In the second trial (Fig. 6), no adverse events were observed in bison following oral vaccination with BCG or HIMB. Furthermore, none of the animals exhibited fever or respiratory distress after aerosol challenge with *M. bovis* 0286 and remained in good condition till the end of the study. While all BCG vaccinated bison were found to weigh significantly more than mock vaccinated animals at 6-weeks post-challenge (wpc), by 13-wpc this difference had diminished as even mock vaccinated animals had started to gain weight (Fig. 7). Although some HIMB vaccinated bison were found to weigh more than mock vaccinated animals at both 6- and 13-wpc, the gains were not as high as in BCG vaccinated animals (Fig. 7).

### DTH response

**Fig. 8:**
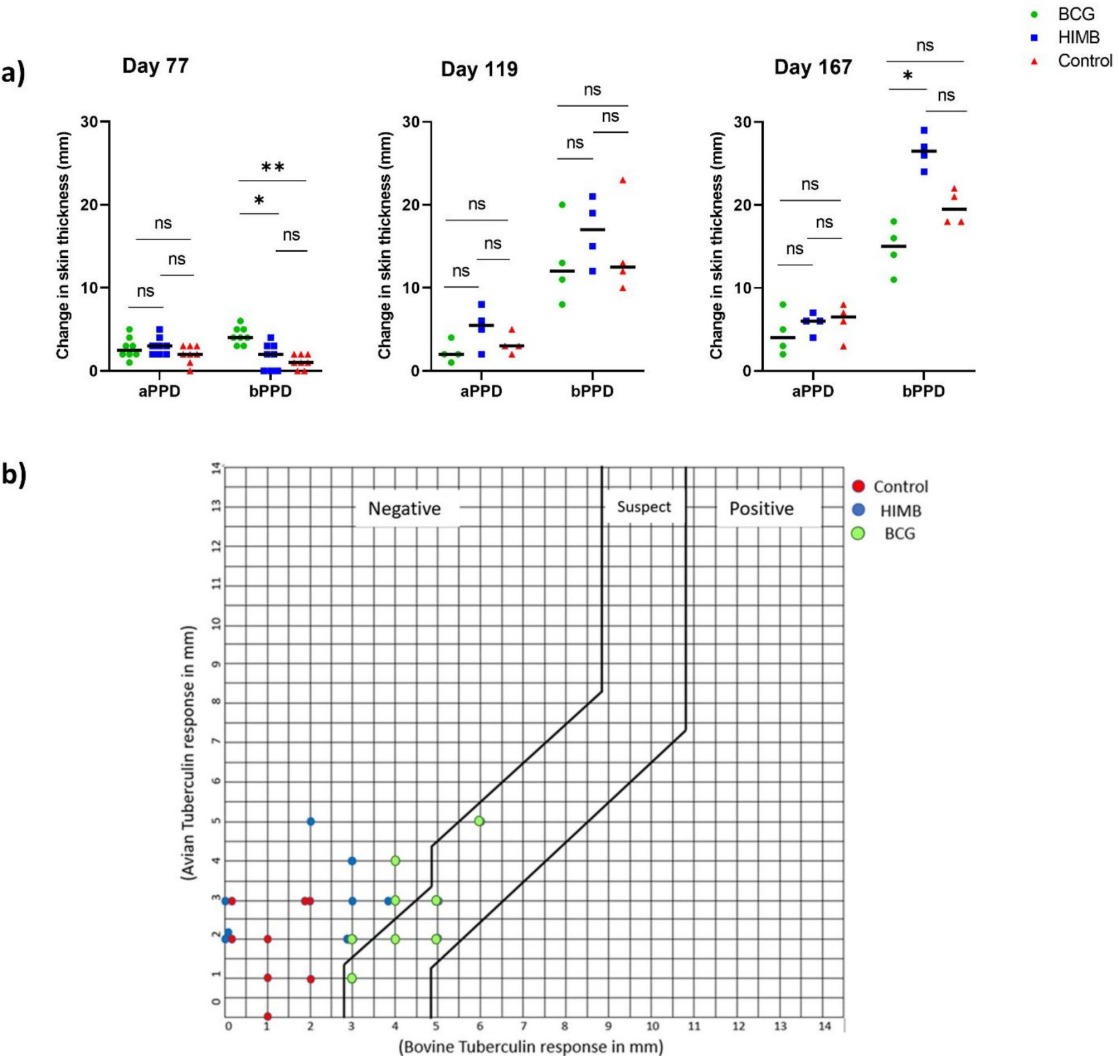
Tuberculin sensitivity in vaccinated and unvaccinated bison. (**a**) Comparative cervical skin test on day 77, 119 and 167 post vaccination, expressed as change in skin thickness (in mm) 72 h after intradermal injection of avian PPD and bovine PPD. Each dot represents change in skin thickness of individual animal and horizontal bar is the median. Bovine PPD elicited significantly greater response in BCG-vaccinated calves compared to HIMB-vaccinated and control calves on day 77. However, the skin thickness was significantly greater in HIMB-vaccinated calves relative to the BCG-vaccinated calves on day 167. (**b**) Comparative cervical test results of control, HIMB and BCG vaccinated animals at 77 days post-vaccination shown in a matrix where X-axis represents bPPD response (change in skin thickness in mm) and Y-axis represents aPPD response (change in skin thickness in mm). Each dot on the graph indicates response of an individual animals to both avian and bovine tuberculin. The three zones in the graph from left to right indicate negative, suspected, and positive for Bovine TB. This matrix is based on Canadian Food Inspection Agency’s interpretation criteria for Comparative Cervical Tuberculin Test (Form 1645). Statistical analysis using Kruskal Wallis Test with Dunn’s multiple comparison where ** indicates significance at *p* < 0.01 and * indicates significance at *p* < 0.05.

CCT measurements done on all animals 77 days after administration of the first vaccine dose but just prior to aerosol challenge with virulent *M. bovis* 0286 revealed that the skin thickness changes in bison given BCG were significantly greater than in animals given HIMB and animals in the mock vaccine control group (Fig. 8a). Most notably, skin thickness changes in animals given HIMB and animals in the mock vaccine control group were not different (Fig. 8a). These results are also reflected in the CCT graph based on the Canadian Food Inspection Agency CCT interpretation matrix (CFIA form 1645). Based on the skin thickness change measurements, all mock vaccinated and HIMB animals fall in the negative zone while five out of eight animals given BCG fall in the suspect zone of the CCT graph (Fig. 8b)^34^.

As expected, CCT measurements at 6- and 13-wpc (or 119 and 167 days after the first vaccine dose) yielded increased skin thickness changes in all animals indicating BTB-associated DTH to bPPD. Notably, at 13 wpc, HIMB vaccinated and *M. bovis* challenged animals had significantly higher DTH response relative to BCG vaccinated and *M. bovis* challenged animals. (Fig. 8a).

### Gross Pathology

**Fig. 9:**
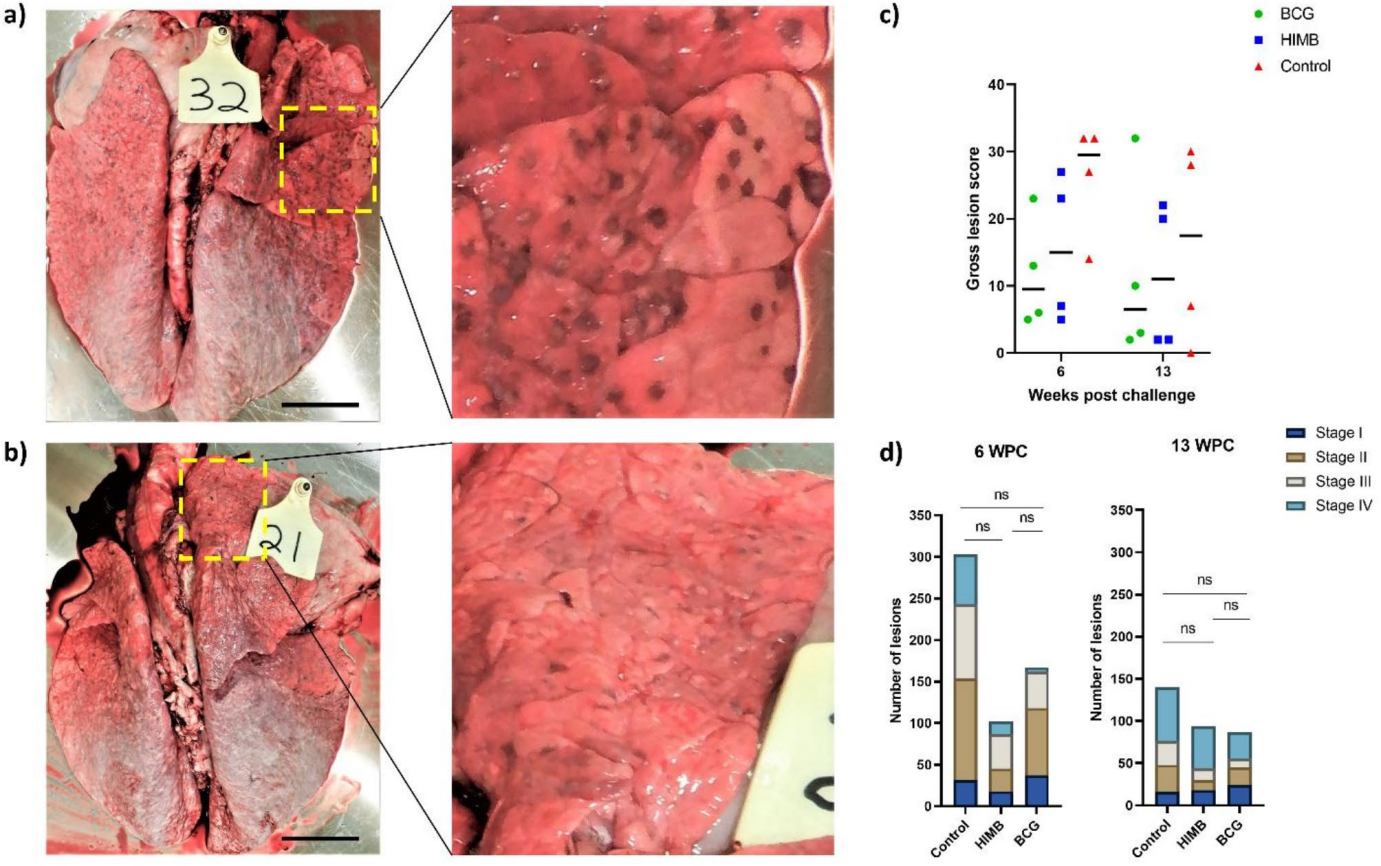
Characteristics of tuberculous lung lesions and histopathology in vaccinated and unvaccinated bison challenged with *M. bovis* 0286. (**a**) Lungs of mock-vaccinated control animals with numerous tubercles visible on the surface of the lungs (inset). (**b**) Lungs of BCG-vaccinated calves showing very few lung lesions (inset). Scale bar = 75 mm. (**c**) A cumulative gross lung lesion score from cranial, middle, caudal and accessory lung lobes. Each data point represents total lesion score of an individual animal from the control, HIMB and BCG vaccinated groups and the horizontal line indicates group median. The overall lesion score decreased in calves euthanized at 13 weeks post challenge compared to the calves euthanized earlier at 6 weeks post challenge. No statistical difference was noted between treatment groups or across the two euthanasia points (ANOVA, *p* = 0.131 and 0.856 respectively). (**d**) Enumeration of the microscopic granulomas in the lung samples of bison calves vaccinated with BCG, HIMB and a mock control followed by challenge with *M. bovis*. Statistical analysis using Kruskal Wallis Test with Dunn’s multiple comparison revealed no significant difference.

Visual assessment at necropsy and palpation of the individual lung lobes and cut sections revealed BCG and HIMB vaccinated bison had fewer lung lesions compared to mock vaccinated animals. Indeed, almost all of the animals in the mock vaccinated group presented with distinct and numerous multifocal to coalescing nodules in the lungs (Fig. 9a) compared to BCG vaccinated animals (Fig. 9b). Overall, BCG vaccinated bison had the lowest median lung lesion scores followed by HIMB vaccinated animals (Fig. 9c). BTB-associated lesions were also observed in the mediastinal, tracheobronchial, and retropharyngeal LNs from most of the animals. Qualitatively however, there were fewer areas of LN tissue containing necrotic material in both BCG and HIMB vaccinated animals compared to mock vaccinated animals (data not shown).

### Histopathology

Microscopic granulomatous lesions were observed in all animals from this trial, and a large number of acid-fast mycobacteria were also observed in the ZN sections of lung tissues from mock vaccinated animals. At 6-wpc, there were 66% and 45% less lesions observed in HIMB and BCG vaccinated bison respectively compared to the mock vaccinated animals (Fig. 9d). By 13-wpc, the reduction in the total number of microscopic lung lesions due to HIMB vaccination was 33% and that due to BCG vaccination was 38% compared to mock vaccinated animals (Fig. 9d). However, the proportion of stage IV granulomatous lesions was the lowest in the lungs of BCG vaccinated animals at both 6- and 13-wpc (Fig. 9d). Notably, the total number of lesions also appeared to trend downwards over time in mock vaccinated animals.

### Bacterial burden

**Fig. 10:**
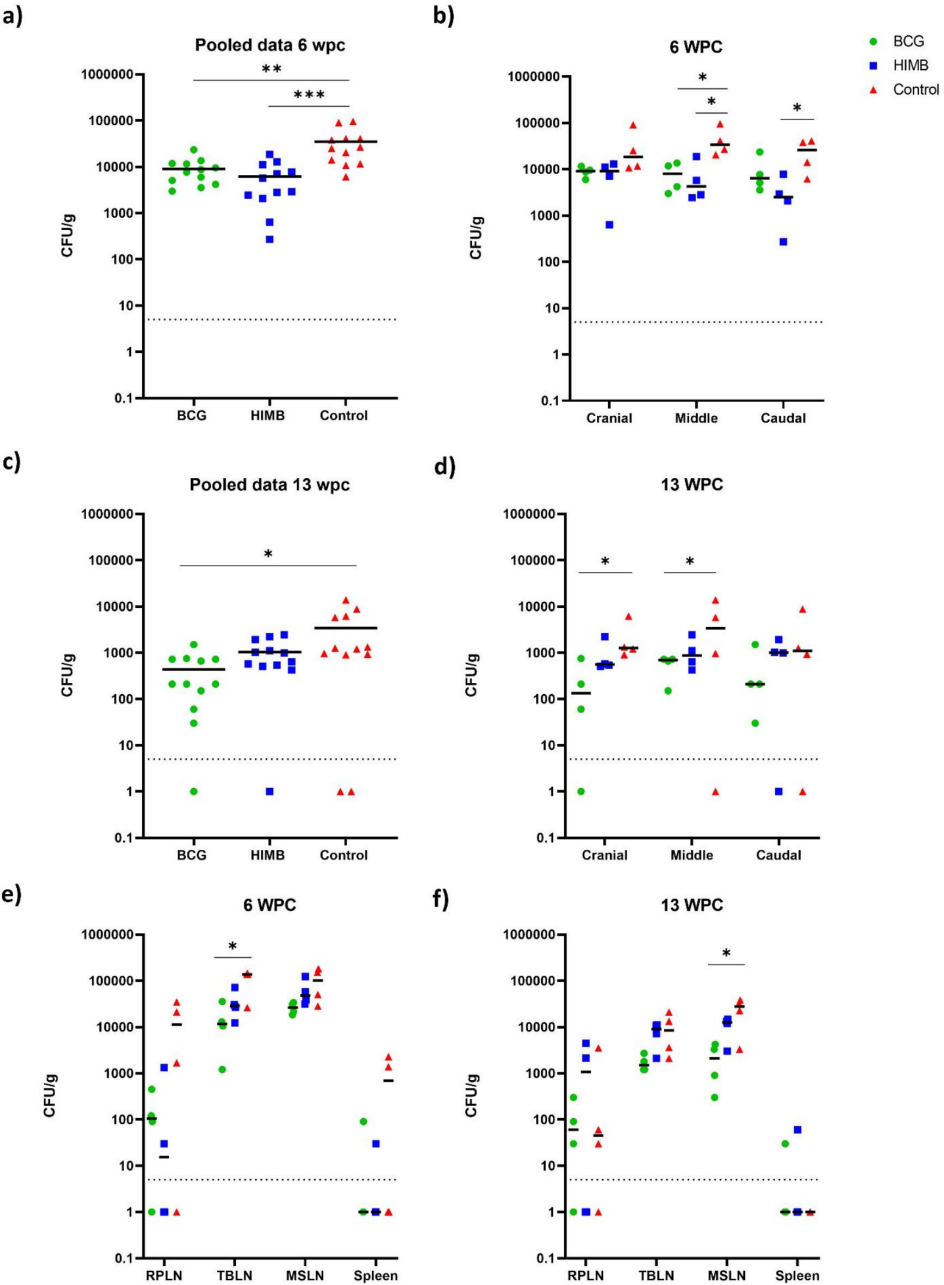
Bacterial burden in the lungs and lymphoid tissues of vaccinated and unvaccinated bison challenged with *M. bovis* 0286. Each dot represents CFU/g of lung tissue from an animal and the horizontal bar represent the median. (**a**) At 6 weeks post challenge, significantly fewer bacteria were isolated from the middle and caudal lung lobes of BCG- and HIMB-vaccinated calves as compared to the unvaccinated controls. (**b**) Likewise, pooled data from all the lung lobes revealed significantly reduced bacterial load in BCG-vaccinated and HIMB-vaccinated calves. (**c**) At 13 weeks post challenge, significantly fewer bacteria were isolated from the cranial and middle lung lobes of BCG-vaccinated calves as compared to the unvaccinated controls. (**d**) Similarly, pooled data from all the lung lobes revealed significantly reduced bacterial load in BCG-vaccinated calves relative to the controls. (**e**) Bacterial load in the lymphoid tissues was qualitatively lower in the BCG and HIMB-vaccinated calves at both 6 weeks (**e**) and 13 weeks (f) post challenge. In fact, a significant reduction in CFU in the BCG-vaccinated group was observed in the tracheo-bronchial LN and mediastinal LN at 6 and 13 weeks respectively. Statistical analysis using unpaired T-test (for figures a, c, e and f) and one-way ANOVA with Tukey multiple comparison (for figures b and d) where *** indicates significance at *p* < 0.001, ** indicates significance at *p* < 0.01 and * indicates significance at *p* < 0.05.

Overall, the burden of live *M. bovis* 0286 was significantly lower in the lungs of bison administered either BCG or HIMB than in mock vaccinated animals at 6-wpc (Fig. 10a). At this time-point, *M. bovis* 0286 burden in the middle and caudal lung lobes only of BCG and HIMB vaccinated animals were lower compared to mock vaccinated controls (Fig. 10b). By 13-wpc, viable *M. bovis* 0286 burden in all lung lobes of BCG vaccinated bison were significantly less than in mock vaccinated animals (Fig. 10c). This was not seen in HIMB vaccinated animals (Fig. 10c). At 13-wpc, there was a significantly lower bacterial burden in the cranial and middle lung lobes of BCG vaccinated animals relative to the controls (Fig. 10d). Interestingly, we observed that in all animals including the mock vaccinated ones, the overall burden of *M. bovis* 0286 decreased by approximately10-fold in the time-period between 6- and 13-wpc.

Live *M. bovis* 0286 was also recovered from several lymphoid tissues, specifically the retropharyngeal LNs, tracheobronchial LNs, mediastinal LNs and spleens from vaccinated as well as mock vaccinated bison (Fig. 10e). Compared to mock vaccinated animals, the *M. bovis* 0286 burden at 6-wpc was significantly lower in all lymphoid tissues including in the spleens of BCG vaccinated bison and with the exception of the spleen stayed that way even by 13-wpc (Fig. 10f). This was not consistently seen in HIMB vaccinated animals.

## Discussion

Although it was demonstrated that bison can be experimentally infected with *M. bovis*^26^, it remained unknown if they develop and present clinical signs associated with active BTB disease. Furthermore, the bison in the aforementioned study were challenged with live *M. bovis* via the intratracheal instead of the aerosol route of infection, and viable *M. bovis* burdens in the lungs and lymph nodes at different timepoints post-challenge were not quantified to determine *M. bovis* replication. Our study addresses these missing points.

In the first part of our study, we found that bison challenged with aerosolized *M. bovis* 0286 at high (1 × 10^6^ CFUs/animal) and at low (1 × 10^4^CFUs/animal) doses to mimic airborne infection^35^ failed to thrive, became sensitized to bPPD, developed lung lesions, and burdened with viable TB bacilli in key organs. These results are consistent with findings in other animal models that were also aerosol challenged with *M. bovis* and developed BTB^36,37^. Although fever and respiratory distress was not observed in any of the challenged bison, more than 40% of the animals including those given high (HD) and low dose (LD) *M. bovis*, failed to thrive and even lost weight by the end of the trial. This is also consistent with our previous observations of domestic pigs, another large animal model, that were also aerosol challenged with similar doses of *M. bovis*^38^. The comparative cervical skin test (CCT) is an established method to diagnose BTB in livestock and some wildlife species^13,39^. In this study, all experimentally challenged bison tested positive by CCT with DTH responses to bPPD in both HD- and LD infected- bison being similar. While we show CCT works well in bison, the need to capture free-ranging wildlife species like wood bison twice, once to administer tuberculin and the second time 72-hours later to examine and measuring for changes in skin thickness is extremely cumbersome and would be highly impractical in WBNP^40^. As such, alternative ways to diagnose BTB in bison at a single time-point with high sensitivity and specificity in the field would be highly desirable^41,42^ and is the focus of our ongoing work. One of the approaches that is feasible as an ante-mortem test for free-ranging bison is the use blood-based interferon gamma release assay (IGRA).

We noted that bison challenged with HD and LD *M. bovis* developed BTB lesions in the lungs and lymph nodes, an outcome that is similar to what is observed in cattle aerosol challenged with *M. bovis*^37,43,44^. For example, we detected pulmonary lesions in all 6 animals euthanized early at day 31 post-challenge. This is consistent with reports of early granuloma formation in cattle challenged with a field strain of *M. bovis*^30^. However, the severity of lesions was visibly greater in HD *M. bovis* bison and evidenced by higher lesion scores as well as the detection of more advanced stage microscopic granulomatous lesions in the lungs of these animals. Compared to LD *M. bovis* bison, the burden of live *M. bovis* in the lungs of HD *M. bovis* bison was also significantly higher and stayed that way over time post-challenge. Interestingly, we noticed that *M. bovis* burden in the lungs of LD *M. bovis* bison but not HD *M. bovis* bison trended downwards over time. This suggests that host immunity-mediated control of the pathogen is possible up to a certain extent if the initial challenge dose is not overwhelming.

Live BCG given orally to livestock and various wildlife species has been shown to protect against BTB^12,16,19–24,45^. Likewise, heat-inactivated killed *M. bovis *has been found to protect cattle^46^, deer^24^, goats^47^, badgers^16^and wild pigs^48^ against BTB. Therefore, we reasoned oral vaccination of a free-ranging species like bison with either BCG or heat-inactivated *M. bovis* might be the best approach to take towards BTB control in WBNP. To test this, in the second part of this study we examined and compared the protective efficacy of orally administered live BCG and heat-inactivated *M. bovis* (HIMB) using our bison BTB model. While both BCG and HIMB were found to offer protection against BTB, the live attenuated BCG vaccine is more efficacious in bison. Specifically, animals given BCG and then challenged with virulent *M. bovis* gained more weight, presented with significantly fewer lung lesions and granulomas, and much reduced *M. bovis* burden in lungs and lymphoid tissues compared to HIMB and mock vaccinated animals. Moreover, detection of the lowest proportions of stage IV granulomatous lesions in the lungs of BCG vaccinated animals at both 6- and 13-wpc was strongly indicative of vaccine-mediated *M. bovis* control. While the protective efficacy of the HIMB vaccine did not appear to be as high as that offered by BCG, bison administered the killed vaccine nonetheless thrived and presented with qualitatively fewer lung lesions and granulomas than mock vaccinated animals. Notably, *M. bovis* burden in the lungs of HIMB vaccinated animals at 6-wpc but not 13-wpc, was lower than in mock vaccinated animals. This suggests the protection offered by HIMB may not be as durable as that offered by BCG. One plausible reason for the differences in protective efficacy between BCG and HIMB could be due to live BCG being able to persist in a vaccinated host longer than HIMB. Another plausible reason may be the higher dose of BCG at 1 × 10^8^ CFUs/animal versus HIMB at 1 × 10^7^ CFUs/animal given at the prime vaccination time-point. Furthermore, we noted again that the overall *M. bovis* burden in the lungs of mock vaccinated bison decreased over time. This supports our notion that host immunity-mediated control of *M. bovis* can occur to some extent.

Given that live BCG has in common with *M. bovis *many of the proteins found in tuberculin and that its administration sensitizes the host to bovine tuberculin or bPPD^28^, a significant drawback with BCG use is that the ability to differentiate vaccinated from infected animals is lost when the tuberculin skin test is performed^49^. Indeed, the CCT results of bison administered oral BCG in this study is consistent with this notion. In contrast, we found that oral administration of HIMB did not sensitize bison to bovine tuberculin. This observation is consistent with what has also been reported regarding HIMB use in cattle^28^, goat^50^, deer^24^, and wild boar^23,48^. While the mechanism underlying the lack of response to bovine tuberculin in oral HIMB vaccinated animal is not clear, the denaturation of antigenic epitopes during heat-inactivation and/or the oral route of administration of HIMB may be possible explanations for this effect^28,51^. Most importantly, our findings suggest the use of HIMB in bison could still allow the use of existing bovine tuberculin based BTB tests and enable the differentiation of infected from vaccinated animals.

Sensitization to bovine tuberculin notwithstanding, our results clearly indicate oral live BCG is a superior option for controlling BTB in bison, in much the same manner as in cattle^52,53^. Moreover, there is strong evidence for the utility of oral BCG in protecting animals at low population densities^54^against BTB compared to populations with high animal-to-animal contact^52^. The potential utility of this vaccine in bison is further bolstered by a recent report that BCG vaccination significantly reduces transmission of BTB in cattle^55^. Furthermore, challenges with respect to sensitization to bovine tuberculin might be overcome with the use of defined *M. bovis*-specific antigens in diagnostic tests for BTB in BCG-vaccinated bison^56,57^. Moreover, blood based diagnostic test such as Interferon gamma release assay using defined antigens like DST-F (ESAT-6, CFP-10, Rv3615c) could be used instead of conventional skin test to accurately diagnose bovine TB in bison^58^. This further obviates the need to re-capture the animals unlike for skin test.

Even with all of the factors above being taken into consideration, conclusive evidence of the benefits of either live BCG or HIMB oral vaccination in protecting free-ranging wildlife species against BTB will ultimately only be obtained through large scale, longitudinal trials in naturally infected populations^59^. In the case of WBNP wood bison, such efforts are certainly warranted given our promising findings.

## Materials and methods

### Ethics Statement

All the methods used in this study were carried out in accordance with relevant guidelines and regulations set by the Animal Research Ethics Board of the University of Saskatchewan (Animal Use Protocol # 20190085). In addition, all the methods used in the study are reported in accordance with Animal Research: Reporting of In Vivo Experiments (ARRIVE) guidelines. All experimental procedures and methods were performed in accordance with applicable regulations and guidelines.

### Experimental design

In the first trial (Fig. 1), eighteen 6-month-old bison housed at the University of Saskatchewan’s Livestock and Forage Centre of Excellence (LFCE) were ear-tagged and randomly assigned into two groups of nine animals each. Each animal was also weighed before being transported to the CL3-Ag facility at Vaccine and Infectious Disease Organisation (VIDO). After 1 week of acclimatization, the animals were aerosol challenged with either a high dose (HD: 1 × 10^6^ CFUs/animal) or a low dose (LD: 1 × 10^4^ CFUs/animal) of virulent *M. bovis* and monitored daily thereafter. Comparative cervical skin tests (CCT) were performed only on three randomly picked animals from each treatment group at three different timepoints of 32-, 45- and 60-days post-challenge (dpc) and just prior to their euthanasia and necropsy. All animals were weighed at euthanasia. Euthanasia was carried out by the intravenous injection of sodium pentobarbital.

In the second trial (Fig. 6), twenty four 6-month-old bison housed at the LFCE were ear-tagged and randomly assigned into three groups of eight animals each. Each animal was weighed before being transported to the CL3-Ag facility at VIDO. Animals in each group were then orally administered either saline, heat-inactivated *M. bovis* (HIMB) or BCG Danish as illustrated (Fig. 6) in a prime-boost regimen 28 days apart. CCT was performed on all animals 75 days after the first vaccination and just prior to challenge with 1 × 10^5^ CFUs/animal of virulent *M. bovis*. After challenge however, CCT was performed only on four randomly picked animals from each treatment group prior to their euthanasia and necropsy. All animals were weighed at euthanasia. Euthanasia was carried out by the intravenous injection of sodium pentobarbital.

### Preparation of challenge material

A Wood Buffalo National Park *Mycobacterium bovis* strain (CFIA 2015TB0286) isolated from a diseased bison (referred to as *M. bovis*0286) was obtained from the Canadian Food Inspection Agency (CFIA). This strain has been sequenced and well characterized by the CFIA^60^. It was cultured in 7H9 liquid media supplemented with 10% Albumin Dextrose Catalase (ADC), 0.5% sodium pyruvate and 0.05% Tween-80 with agitation at 37^°^C until mid-log phase of growth. Several 1 mL aliquots of this were prepared and frozen. A few days before the challenge, a frozen aliquot was taken, sub-cultured into a fresh complete 7H9 liquid media and grown with agitation at 37^°^C. On the day of the challenge, optical density (OD at 600 nm) was measured, and an appropriate volume of this culture was added to saline to obtain the required doses of the challenge inoculum. For the first trial, a high dose (5 × 10^5^ CFU/ml) and a low dose (5 × 10^3^ CFU/ml) inoculum were prepared such that challenge with 2 ml of the inoculum corresponds to 1 × 10^6^ CFU/animal for high dose and 1 × 10^4^CFU/animal for the low dose. For aerosol challenge, inoculums were transferred into a jar attached to a Collison nebulizer (CH Technologies, USA), connected to a tank of compressed oxygen. Aerosol generated was passed through a tube into a mask that fitted snugly to the bison muzzle. The flow rate of the nebulizer was adjusted to 1 ml per minute and therefore each bison calve was exposed to aerosol challenge for 2 min^38^. The animals were not sedated for the procedure but were restrained in a mechanical squeeze inside the Biosafety Level 3 containment rooms. Aliquots of the challenge inoculums were taken, serially diluted, plated on 7H11 agar supplemented with pyruvate and cultured at 37 °C for 4 weeks. CFU count suggested delivery of close to 1 × 10^6^ CFUs/animal (HD) and 1 × 10^4^ CFUs/animal (LD) in trial 1 and 1 × 10^5^ CFUs/animal in trial 2.

### Vaccine Preparation and Administration

For both the prime and boost doses of live BCG, BCG-Danish 1331 was grown in 7H9 media with 10% Albumin Dextrose Catalase (ADC) and 0.05% Tween-80 to mid-logarithmic phase of growth. OD_600nm_ was measured and an aliquot of the culture was added to saline so that an estimated cell density of 2 × 10^6^ CFUs/mL might be attained. It was estimated that if 5 mL of this BCG in saline were to be administered by gavage to each bison, every animal would receive 10^7^CFUs of live BCG at prime and boost time-points. This choice of dose was based on previous studies^28,54^. To verify cell densities, aliquots of the BCG in saline mixture were taken, serially diluted, plated on 7H11 agar and cultured at 37 °C for 4 weeks while the rest of the live BCG in saline was orally administered to bison by gavage. CFU counts of the live BCG in saline revealed the prime BCG dose was approximately 1.5 × 10^7^ CFUs/mL while the boost dose was approximately 1.8 × 10^6^ CFUs/mL. Thus, each bison in the BCG group actually received 0.75 × 10^8^ CFUs of BCG at prime and about 10^7^ CFUs of BCG at boost.

For both the prime and boost doses of HIMB, *M. bovis* 0286 was grown in 7H9 media with 10% Albumin Dextrose Catalase (ADC), 0.5% sodium pyruvate and 0.05% Tween-80 to mid-logarithmic phase of growth. OD_600nm_ was measured and an aliquot of the culture was added to saline so that an estimated cell density of 2 × 10^6^ CFUs/mL might be attained. An aliquot of the live *M. bovis* 0286 in saline was taken, serially diluted, plated on 7H11 agar supplemented with pyruvate and cultured at 37 °C for 4 weeks for CFU enumeration. The rest of the *M. bovis* 0286 in saline was centrifuged and the cell pellet resuspended in small volume of saline which was then transferred into air-tight microcentrifuge tubes. Subsequently, the tubes were placed in a heat-block set at 85^0^C and inactivated for 45 min as mentioned elsewhere^16,61^. Heat inactivated *M. bovis* was made up to the final required volume with saline and orally administered to bison by gavage. Each animal was given 5mL of the heat inactivated *M. bovis* at 2 × 10^6^ CFU/mL. Several aliquots of the heat-treated vaccine preparation were plated in 7H11 plates to confirm inactivation. CFU counts of the live *M. bovis* 0286 in saline prior to heat-inactivation revealed the prime and boost doses were 1.95 × 10^6^ and 1.89 × 10^6^ CFUs/mL respectively. Thus, each bison likely received very close to 10^7^ CFUs of HIMB at prime and boost.

### Comparative cervical skin-test (CCT)

A region in the neck was shaved and 0.1 mL each of bovine purified protein derivative (bPPD) and avian purified protein derivative (aPPD) were injected intradermally. Both bPPD and aPPD were obtained from the Canadian Food Inspection Agency (CFIA). The concentration for bovine and avian tuberculins were 30,000 IU/mL and 25,000 IU/mL respectively. Skin thickness at the injection sites were measured using Vernier calipers before and 72 h after the administration of the PPDs. A positive skin test was indicated if the increase in skin thickness at the injection site of bovine PPD after 72 h exceeded 4 mm compared to the increase in skin thickness at the injection site of avian PPD^1^. In the first trial, CCT was performed at 3 different timepoints post challenge on days 32, 45 and 60. Likewise, in the second trial, CCT was done on days 77, 119 and 167. The reading of skin thickness was planned to coincide either with blood collection or euthanasia to ensure minimum handling of the animals.

### Pathology

The presence of gross tuberculous lesions was determined by observation and palpation of the entire lung. Each lung lobe was assigned a score based on the number and size of the tuberculous lesions present. The final lung lesion score for each animal was obtained by adding up the lesion scores of all the lung lobes^38,44^.

For histopathological evaluation, small section of tissues was dissected and placed in a tissue cassette, immersed into 10% buffered formalin, and kept at room temperature in BSL-3 to allow fixation. Tissue sections were collected from all the lung lobes, medial and lateral retropharyngeal lymph nodes (LN), tracheobronchial LN, mediastinal LN, and spleen. The formalin-fixed sections were then processed at Prairie Diagnostic Services (PDS) for Hematoxylin-Eosin (H&E) and Ziehl-Neelsen (ZN) staining. Granulomatous lesions were categorized into one of the 4 stages following established methods^32,33^. Stage 1 (initial): irregular unencapsulated clusters of epithelioid macrophages, lymphocytes, and small numbers of neutrophils. Stage 2 (solid): Epithelioid macrophages were enclosed by a thin capsule, with infiltration of lymphocytes, neutrophils and often Langhan’s multinucleated giant cells. Stage 3 (minimal necrosis): The granuloma is fully encapsulated with fibrous capsule, with central necrotic areas, which were caseous and mineralized. Stage 4 (necrosis and mineralization): Thickly encapsulated, large, irregular, multicentric granulomas with prominent caseous necrosis, extensive mineralization occupying the greater part of the lesion.

### Tissue bacterial burden estimation

Approximately 1 g of tissue was obtained using a sterile punch biopsy sampler on at least three different lobes of the lungs and one section of different lymph nodes. Sampling was random and sections from visually uninvolved sections of the lungs and lymph nodes were included. Biopsy punch samples were stored in 3 mL of PBSA supplemented with Tween-80 (0.05%), ampicillin (80 µg/ml) and cycloheximide (100 µg/ml) and homogenized. Dilutions of the homogenate were plated on commercial 7H11 selective plates containing antibiotics and antimycotics (Middlebrook 7H11 selective plates, Hardy Diagnostics). The bacterial colonies were enumerated with the aid of an automated colony counter (Scan 300, InterScience). The limit of detection was 5 CFUs and any count below 5 was replaced by 1 to enable graphical representation on a log scale.

### Statistical analysis

Statistical tests and graphical representation were performed using GraphPad Prism. At the outset, data distribution for each dataset was checked for normality using Kolmogorov-Smirnov (KS) Test. Subsequently, parametric analysis was employed for normally distributed data and non-parametric tests were employed for data that were not distributed normally.

## Electronic supplementary material

Below is the link to the electronic supplementary material.

Supplementary Material 1

Supplementary Material 2

Supplementary Material 3

## Data Availability

The datasets generated during the current studies are sensitive and will be made available from the corresponding author only on reasonable request.
