# Supplementary material for: Orally administered live BCG and heat-inactivated Mycobacterium bovis protect bison against experimental bovine tuberculosis
Source: Sci Rep. 2025 Jan 30;15:3764. doi: 10.1038/s41598-025-88176-0 (PMC11782570; doi:10.1038/s41598-025-88176-0)
Supplement: Supplementary file 1 — Supplementary Material 1 [file 41598_2025_88176_MOESM1_ESM.docx]

Supplementary Table 1 (S1): Skin thickness measurement post-vaccination with BCG and HIMB

| **Animal ID** | **PPDA(0hr)** | **PPDA(72hr)** | **change** | **PPDB(0hr)** | **PPDB(72hr)** | **change** | **Group** | **Sex** |
| --- | --- | --- | --- | --- | --- | --- | --- | --- |
| 20b | 5 | 7 | 2 | 4 | 8 | 4 | BCG | F |
| 21 | 5 | 8 | 3 | 5 | 10 | 5 |  | M |
| 29 | 4 | 8 | 4 | 4 | 8 | 4 |  | M |
| 30 | 5 | 10 | 5 | 4 | 10 | 6 |  | M |
| 31 | 7 | 8 | 1 | 6 | 9 | 3 |  | M |
| 36 | 6 | 8 | 2 | 6 | 9 | 3 |  | M |
| 37 | 6 | 9 | 3 | 6 | 10 | 4 |  | M |
| 39 | 5 | 7 | 2 | 4 | 9 | 5 |  | F |
| 23 | 5 | 7 | 2 | 5 | 5 | 0 | HIMB | M |
| 26 | 5 | 10 | 5 | 4 | 6 | 2 |  | M |
| 34 | 3 | 7 | 4 | 3 | 6 | 3 |  | F |
| 35 | 4 | 7 | 3 | 4 | 8 | 4 |  | F |
| 38 | 4 | 7 | 3 | 3 | 6 | 3 |  | M |
| 40 | 5 | 7 | 2 | 5 | 5 | 0 |  | F |
| 41 | 4 | 7 | 3 | 6 | 6 | 0 |  | M |
| 42 | 5 | 7 | 2 | 4 | 6 | 2 |  | F |
| 19 | 4 | 5 | 1 | 4 | 6 | 2 | Control | M |
| 22 | 5 | 8 | 3 | 4 | 6 | 2 |  | M |
| 24 | 4 | 7 | 3 | 5 | 5 | 0 |  | M |
| 25 | 5 | 7 | 2 | 5 | 5 | 0 |  | M |
| 27 | 5 | 5 | 0 | 4 | 5 | 1 |  | M |
| 28 | 5 | 8 | 3 | 3 | 5 | 2 |  | M |
| 32 | 5 | 7 | 2 | 6 | 7 | 1 |  | F |
| 33 | 4 | 6 | 2 | 4 | 5 | 1 |  | F |
