# Supplementary material for: Orally administered live BCG and heat-inactivated Mycobacterium bovis protect bison against experimental bovine tuberculosis
Source: Sci Rep. 2025 Jan 30;15:3764. doi: 10.1038/s41598-025-88176-0 (PMC11782570; doi:10.1038/s41598-025-88176-0)
Supplement: Supplementary file 2 — Supplementary Material 2 [file 41598_2025_88176_MOESM2_ESM.docx]

Supplementary Table 2 (S2): Skin thickness measurement 6 weeks post-challenge with *M. bovis*

| **Animal ID** | **PPDA(0hr)** | **PPDA(72hr)** | **change** | **PPDB(0hr)** | **PPDB(72hr)** | **change** | **Group** | **Sex** |
| --- | --- | --- | --- | --- | --- | --- | --- | --- |
| 21 | 7 | 11 | 4 | 6 | 17 | 11 | BCG | M |
| 29 | 8 | 9 | 1 | 7 | 20 | 13 |  | M |
| 37 | 7 | 9 | 2 | 6 | 14 | 8 |  | M |
| 39 | 7 | 9 | 2 | 6 | 26 | 20 |  | F |
| 35 | 7 | 12 | 5 | 7 | 26 | 19 | HIMB | F |
| 40 | 7 | 15 | 8 | 6 | 27 | 21 |  | F |
| 41 | 7 | 9 | 2 | 6 | 18 | 12 |  | M |
| 42 | 5 | 11 | 6 | 5 | 20 | 15 |  | F |
| 19 | 5 | 8 | 3 | 6 | 16 | 10 | Control | M |
| 25 | 6 | 9 | 3 | 7 | 20 | 13 |  | M |
| 27 | 5 | 7 | 2 | 5 | 17 | 12 |  | M |
| 32 | 5 | 10 | 5 | 6 | 29 | 23 |  | F |
