# Supplementary material for: Orally administered live BCG and heat-inactivated Mycobacterium bovis protect bison against experimental bovine tuberculosis
Source: Sci Rep. 2025 Jan 30;15:3764. doi: 10.1038/s41598-025-88176-0 (PMC11782570; doi:10.1038/s41598-025-88176-0)
Supplement: Supplementary file 3 — Supplementary Material 3 [file 41598_2025_88176_MOESM3_ESM.docx]

Supplementary Table 3 (S3): Skin thickness measurement 13 weeks post-challenge with *M. bovis*

| **Animal ID** | **PPDA(0hr)** | **PPDA(72hr)** | **change** | **PPDB(0hr)** | **PPDB(72hr)** | **change** | **Group** | **Sex** |
| --- | --- | --- | --- | --- | --- | --- | --- | --- |
| 20b | 7 | 12 | 5 | 6 | 24 | 18 | BCG | F |
| 30 | 7 | 15 | 8 | 7 | 18 | 11 |  | M |
| 31 | 9 | 12 | 3 | 8 | 22 | 14 |  | M |
| 36 | 10 | 12 | 2 | 9 | 25 | 16 |  | M |
| 23 | 7 | 11 | 4 | 7 | 33 | 26 | HIMB | M |
| 26 | 10 | 16 | 6 | 8 | 35 | 27 |  | M |
| 34 | 7 | 13 | 6 | 7 | 36 | 29 |  | F |
| 38 | 9 | 16 | 7 | 8 | 32 | 24 |  | M |
| 22 | 6 | 9 | 3 | 7 | 29 | 22 | Control | M |
| 24 | 7 | 13 | 6 | 7 | 25 | 18 |  | M |
| 28 | 8 | 16 | 8 | 9 | 30 | 21 |  | M |
| 33 | 5 | 12 | 7 | 5 | 23 | 18 |  | F |
